# Regulatory impacts of PPARGC1A gene expression on milk production and cellular metabolism in buffalo mammary epithelial cells

**DOI:** 10.1080/10495398.2024.2344210

**Published:** 2024-05-24

**Authors:** Seyed Mahdi Hosseini, Ye Tingzhu, Ran Zaohong, Farman Ullah, Aixin Liang, Guohua Hua, Liguo Yang

**Affiliations:** National Center for International Research on Animal Genetics, Breeding and Reproduction (NCIRAGBR), College of Animal Science and Technology, Huazhong Agricultural University, Wuhan, China

**Keywords:** Buffalo, knockdown, mammary epithelial cell, milk, PPARGC1A

## Abstract

The PPARGC1A gene plays a fundamental role in regulating cellular energy metabolism, including adaptive thermogenesis, mitochondrial biogenesis, adipogenesis, gluconeogenesis, and glucose/fatty acid metabolism. In a previous study, our group investigated seven SNPs in Mediterranean buffalo associated with milk production traits, and the current study builds on this research by exploring the regulatory influences of the PPARGC1A gene in buffalo mammary epithelial cells (BuMECs). Our findings revealed that knockdown of PPARGC1A gene expression significantly affected the growth of BuMECs, including proliferation, cell cycle, and apoptosis. Additionally, we observed downregulated triglyceride secretion after PPARGC1A knockdown. Furthermore, the critical genes related to milk production, including the STATS, BAD, P53, SREBF1, and XDH genes were upregulated after RNAi, while the FABP3 gene, was downregulated. Moreover, Silencing the PPARGC1A gene led to a significant downregulation of β-casein synthesis in BuMECs. Our study provides evidence of the importance of the PPARGC1A gene in regulating cell growth, lipid, and protein metabolism in the buffalo mammary gland. In light of our previous research, the current study underscores the potential of this gene for improving milk production efficiency and overall dairy productivity in buffalo populations.

## Introduction

The Peroxisome proliferator-activated receptor gamma coactivator 1-alpha (PPARGC1A, or PGC-1α) is a protein encoded by the PPARGC1A gene, consisting of 797 amino acids, and situated on chromosome 7 of the river buffalo (Bubalus bubalis, BBU7).^1^ The PPARGC1A gene is essential in governing various aspects of cellular energy metabolism, including adaptive thermogenesis, mitochondrial biogenesis, adipogenesis, gluconeogenesis, and glucose/fatty acid metabolism.^2^^,^^3^ Studies have linked the PPARGC1A gene to milk protein^4^ and milk fat yield^5^ in goats and cattle, and it has been identified as a vital regulator of cattle intramuscular fat.^6^ Hosseini et al.^7^ identified this gene as being associated with specific milk production traits, such as milk yield, fat, and protein percentage, in Mediterranean buffalo. This implies its potential use as a marker to aid in buffalo selection.

Milk, a complex emulsion of water, fat, vitamins, proteins, minerals, and carbohydrates, needs to be synthesized within or transported to the mammary gland.^8^ The mammary gland, critical for milk production, is governed by various genes that regulate its function. Milk is fundamental for the development and healthy growth of newborns due to its optimal composition of nutrients during lactation.^9^ Thus, understanding the mechanisms controlling milk production during lactation is paramount in the agricultural and biomedical sectors.

Research indicates that PPARGC1A is expressed in lactating buffalo mammary gland tissue, demonstrating its role in milk synthesis and secretion.^10^ This gene affects the expression of several genes involved in fatty acid metabolism, glucose uptake, and oxidative phosphorylation. Moreover, numerous transcription factors, including Signal transducer and activator of transcription 5 (STAT5) and PRL-induced Janus kinase 2 (JAK2), essential for the induction of most milk protein genes, are also associated with mammary gland development. Sterol regulatory element-binding protein 1 (SREBP1) and peroxisome proliferator-activated receptor gamma (PPAR-γ) notably regulate key genes in lipid metabolism.^11^ PPARGC1A interacts with the PPAR family (including PPAR-γ, PPAR-α, and PPAR-β/δ)^12^ and modulates nuclear respiratory factors (NRFs) and cAMP response element-binding protein (CREB)^13^ activities.

In buffalo mammary glands, the knockdown of PPARGC1A may significantly impact lactation and milk production. A decrease in gene expression could lead to stunted mammary gland development, hampering the synthesis and secretion of milk proteins, such as casein,^14^ altering lipid metabolism, and diminishing energy utilization, critically affecting milk production. Understanding the consequences of reduced PPARGC1A activity in buffalo mammary glands may offer insights into innovative ways to improve milk production efficiency and buffalo breeding strategies.

We hypothesize that the PPARGC1A gene is instrumental in governing the development of the mammary gland and lactation in dairy buffaloes, which could have far-reaching implications for the agricultural industry.

## Materials & methods

### Isolation and identification of buffalo mammary epithelial cells (BuMECs)

Buffalo mammary epithelial cells (BuMECs) were isolated following the methodology established by Xu et al.^15^ Fresh mammary gland tissue was procured from a disease-free lactating buffalo at a slaughterhouse in Wuhan, China, and was excised aseptically approximately 5 cm from the base of a healthy nipple. After rinsing and carefully removing the connective tissue, the tissue was minced into small pieces, around 1 mm. For subsequent assays, cells in the logarithmic phase of growth were cultured in an incubator at 37 °C with 5% CO2. Following a 24-hour incubation period, the samples were washed three times with PBS and supplemented with fresh medium. The attached cells were then digested at 37 °C for 3 minutes. These digested cells were relocated to a new Petri dish to continue growth. Through selective digestion conducted five times, the buffalo mammary epithelial cells were purified. The identification of buffalo mammary epithelial cells was conducted through immunochemical fluorescence to detect the epithelial cell’s unique marker protein keratin 18.

### siRNA was transfected into mammary epithelial cells

SiRNA targeting the PPARGC1A mRNA sequence from buffalo was designed and synthesized, with negative control (NC) provided by Shanghai Jima Industrial Co., Ltd. The sequence for siPPARGC1A was as follows: forward (F) GGGCUUCUCCAAAGCUGAATT and reverse (R) UUCAGCUUUGGAGAAGCCCTT; the NC sequence was: F: UUCUCCGAACGUGUCACGUTT; R: ACGUGACACGUUCGGAGAATT. Before transfection, well-conditioned cells were chosen for subculturing. These cells were then seeded into 24-well culture plates at an approximate density of 1 × 10^4^ cells per well and incubated at 37 °C with 5% CO2 to encourage further growth. Transfection commenced when cell confluency reached 60%, using the Lipofectamine RNAi MAX kit according to the manufacturer’s guidelines with minor modifications specific to the experiment.

### Real-time fluorescence quantitative PCR

Real-time fluorescence quantitative PCR, commonly known as real-time PCR or qPCR, is a laboratory method that allows for the amplification and concurrent quantification of a specific DNA segment. This technique is particularly valuable for assessing the expression levels of select genes in mammary glands to unveil their roles and effects in miscellaneous biological or pathological contexts, including lactation, development, or cancer.

In the study at hand, siRNA against PPARGC1A was introduced into BuMECs, and real-time fluorescence quantitative qPCR was employed to measure the levels of PPARGC1A mRNA. The assays utilized the QIAGEN SYBR Green Master Kit, and measurements were conducted on a Bio-Rad 384 system. Each sample was assessed in triplicate.

Gene expression levels were determined using the 2^-ΔΔCt^ method,^16^ with GAPDH as the reference gene for normalization. Table 1 presents the primer sequences used for quantifying the mRNA expression levels of genes associated with milk production.

**Table 1.** List of primer sequences for determining relative mRNA expression levels of genes related to milk production.

Gene	Primer Sequence	Tm	Product Length
GAPDH	F:GAAGGGTGGCGCCAAGAGGG R: GGGGGCCAAGCAGTTGGTGG	65.58 66.14	142
Bad	F:GAGGATAATGAAGGGACGGAAGAGG R:TAAGTTGCGGGGCGGAAGGT	62.25 64.28	286
FABP3	F: GAACTCGACTCCCAGCTTGAA R:AAGCCTACCACAATCATCGAAG	60.00 58.46	102
P53	F: CGAGCACTGCCTACCAACAC R: TCCATCCAGAGCATCCTTCA	61.29 58.12	153
PPARGC1A	F:GAGGAATACCAGCACGAA R:AACATAAATCACACGGCG	53.97 53.48	132
SREBF1	F: CTGACGACCGTGAAAACAGA R:GACGGCAGATTTATTCAACTT	58.15 54.57	333
STAT5	F: CACCATCATCAGCGAGCAGCA R:TGACAACCACGGGAAGGGACA	63.35 63.67	293
XDH	F: GAAAGACCCTCCAGCCAACATC R:TCAGCAGAGAGAAAGCAAACAAATC	61.20 60.28	264

F: forward primer; R: reverse primer.

### Determination of fatty acid content in cells

After transfection with buffalo mammary epithelial cells for 36 h, the cells were collected by 8890B-5977B GC/MSD GC/MSD of Agilent Technologies Inc. CA (UAS). The content of long-chain fatty acids in cells was detected by gas chromatography-mass spectrometry (GC-MS).

The chromatographic conditions employed an Agilent DB-FastFAME capillary column (20 m x 0.18 mm x 0.2 µm, from Agilent J&W Scientific, Folsom, CA, USA), utilizing high-purity helium as the carrier gas (with a purity of no less than 99.999%). The flow rate was set at 1.0 ml/min, and the inlet temperature was maintained at 250 °C. The mass spectrometry configuration included an electron bombardment ion source (EI), with the ion source temperature at 230 °C, quadrupole temperature at 150 °C, transfer line temperature at 240 °C, and electron energy at 70 eV.

For data analysis, Masshunter quantitative software by Agilent (USA, version number: V10.0.707.0) was used, which relies on default parameters to automatically detect and integrate all ion fragments, while also facilitating manual verification.

### Cell cycle experiment

To investigate cell cycle distribution in mammary gland cells under conditions where the PPARGC1A gene is knocked down, flow cytometry was utilized to quantify individual cells’ DNA content. This allowed for the determination of the percentage of cells in the G0/G1, S, and G2/M phases. In this study, we performed the cell cycle analysis using a kGI cell cycle detection kit and flow cytometry detection technology, and we strictly followed the kit’s instructions for the operational procedures.

We assessed cells using a flow cytometer to measure their fluorescence values, collecting data from approximately 10,000 cells. The results were then analyzed with ModFit software to compute the number of cells in each cell cycle phase.

### Apoptosis detection

Apoptosis, which is a regulated form of cell death, was detected using the Annexin V-FITC/PI apoptosis kit along with a flow cytometric cell analyzer. Cells in a vigorous growth state were seeded into 6-well culture plates and subjected to the designated treatment when their confluence reached approximately 60%. Subsequently, the fluorescence intensities of Annexin V-FITC and PI in the sample wells were measured, with data collected from about 10,000 cells. The results were then processed using FlowJo software, which facilitated the quantification of the proportion of cells in each apoptotic stage.

### Cell viability assay

Cell proliferation was assessed using the CCK8 (Cell Counting Kit-8) from Tongren Bio, following the protocol provided with the kit. Cells with good growth were plated into 96-well cell culture plates. Upon reaching approximately 60% confluence, the corresponding treatment was performed. After this, the old medium was removed and replaced with a fresh medium mixed with 10% Cell Counting Reagent. The cells were then incubated at 37 °C for 3 hours in the dark. Post-incubation, the absorbance at 450 nm was measured using a fully automated microplate reader.

### Western blot

Western blotting is a widely used molecular biology method to detect and quantify specific proteins in tissue samples, including mammary gland tissue. In this experiment, mammary gland tissue is lysed and homogenized in a buffer containing protease, and phosphatase inhibitors. Consequently, the protein concentration in the lysate is determined using a bicinchoninic acid (BCA) assay. After protein denaturation, 15 µg samples are loaded into wells of a polyacrylamide gel (SDS-PAGE 12%), and an electric current is applied to separate the proteins by size. Subsequently, the separated proteins are transferred from the gel onto a PVDF membrane, and for blocking we used 5% nonfat milk. For immunodetection, the membrane is subjected to incubation with a primary antibody at 4 °C overnight. Quantification of the protein expression used by ImageJ software (National Institutes of Health, Bethesda, MD, United States). To ensure accurate quantification of protein expression levels, we normalize the signals with GAPDH as a housekeeping protein.

### Enzyme-Linked immunosorbent assay

To examine the concentration of β-casein we used a commercial β-casein kit (Mlbio, Shanghai, China). BuMECs were transfected with siPPARGC1A and a cell culture medium was collected. For detecting the β-casein secretion level, we used a 50 µl culture medium. Consequently, we used a microplate reader to measure absorbance at 450 nm (PerkinEnspire, China).

### Statistical analyses

The data were analyzed using SPSS 19.0 (SPSS Inc., Chicago, IL, USA), and graphical representations were generated with GraphPad Prism version 8.0 (GraphPad Software, Inc., La Jolla, CA, USA). For discerning significant differences between two distinct groups, the Student’s t-test was employed. Conversely, differences across multiple groups were evaluated using one-way ANOVA. A P-value of less than 0.05 was designated as the threshold for statistical significance. To guarantee the reliability of the results, all experimental procedures were performed in at least three replicates.

## Results

### mRNA expression levels of related genes after RNAi Treatment

**Figure 1.**
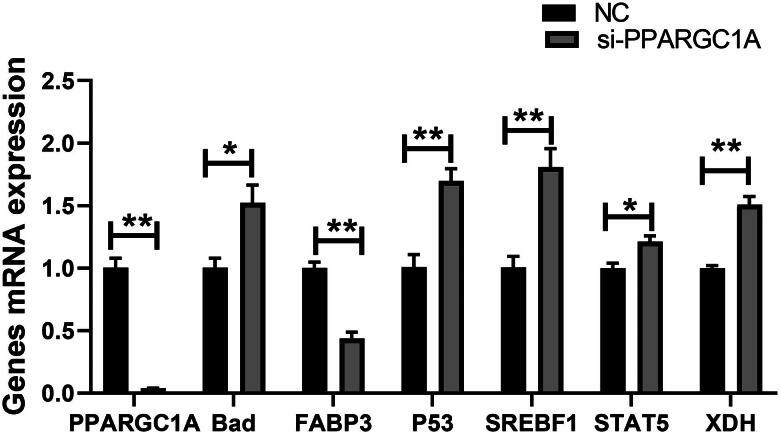
Relative mRNA expression of genes involved in milk production following RNAi Treatment.

The cells underwent siRNA transfection, with the subsequent knockdown confirmed via qRT-PCR. This analysis quantified the impact of PPARGC1A gene suppression on the transcription levels of genes implicated in lactation. The findings suggest that PPARGC1A gene knockdown may influence the expression of related genes (Cell growth, fat, and protein). The RNA interference resulted in a downregulation of the mRNA expression of the PPARGC1A and FABP3 genes (*p* < 0.01), while the silencing of the PPARGC1A gene led to an upregulation of the BAD, P53, SREBF1, STAT5, and XDH genes (*p* < 0.05, *p* < 0.01; Fig. 1).

### Impact of PPARGC1A gene knockdown on the cell cycle of BuMECs

**Figure 2.**
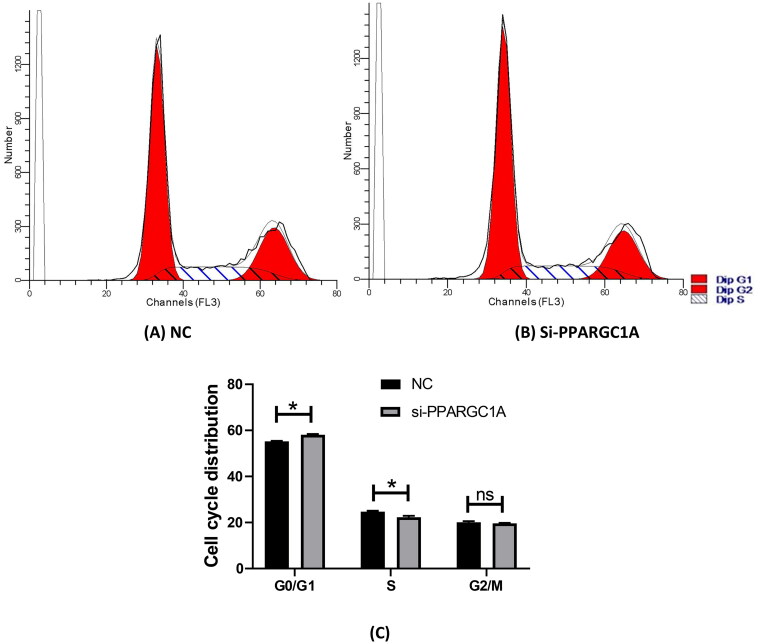
Effect of PPARGC1A gene knockdown on the cell cycle of BuMECs. (A) (NC), and (B) (si-PPARG1A) cell cycle distribution; (C) flow cytometry to detect cell cycle progression.

We assessed the cell cycle distribution of BuMECs using flow cytometry analysis (Fig. 2). This technique measures the DNA content of individual cells, enabling us to ascertain the proportion of cells in the G0/G1, S, and G2/M phases. The study was replicated three times in two distinct groups, comprising a negative control and siPPARGC1A. Following PPARGC1A gene knockdown, there was a pronounced increase in the G0/G1 cell population, while the percentage of cells in the S phase was significantly reduced compared to the control group (*p* < 0.05; Fig. 2). No substantial changes were observed in the G2/M phase.

### Impact of PPARGC1A gene on the viability of BuMECs following RNAi

**Figure 3.**
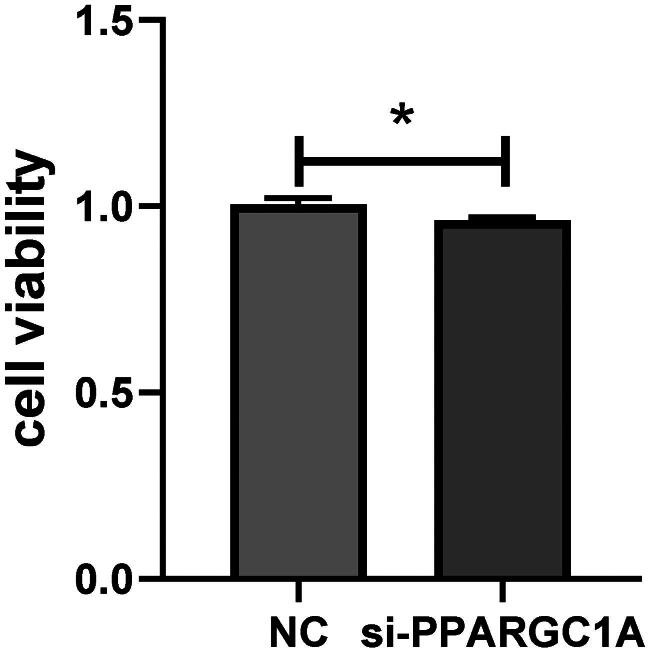
Impact of PPARGC1A gene knockdown on BuMEC viability.

**Figure 4.**
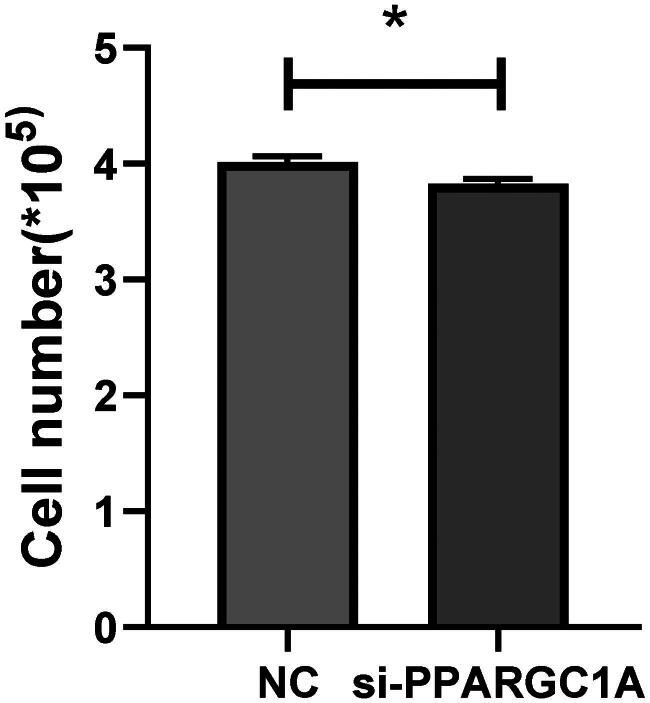
Number of living cells in control and PPARGC1A gene knockdown groups.

We conducted a cell viability assay with five replicates to verify the role of the PPARGC1A gene in cell proliferation. Employing Tongren Bio’s CCK8 kit, we observed a notable impact on the viability of BuMECs following PPARGC1A gene knockdown compared to the respective negative control (NC) groups (*p* < 0.05; Fig. 3). Additionally, cell counts were performed using an automatic cell counter, revealing a significant reduction in cell numbers in the PPARGC1A knockdown cells (*p* < 0.05; Fig. 4).

### Impact of PPARGC1A gene knockdown on apoptosis in BuMECs

**Figure 5.**
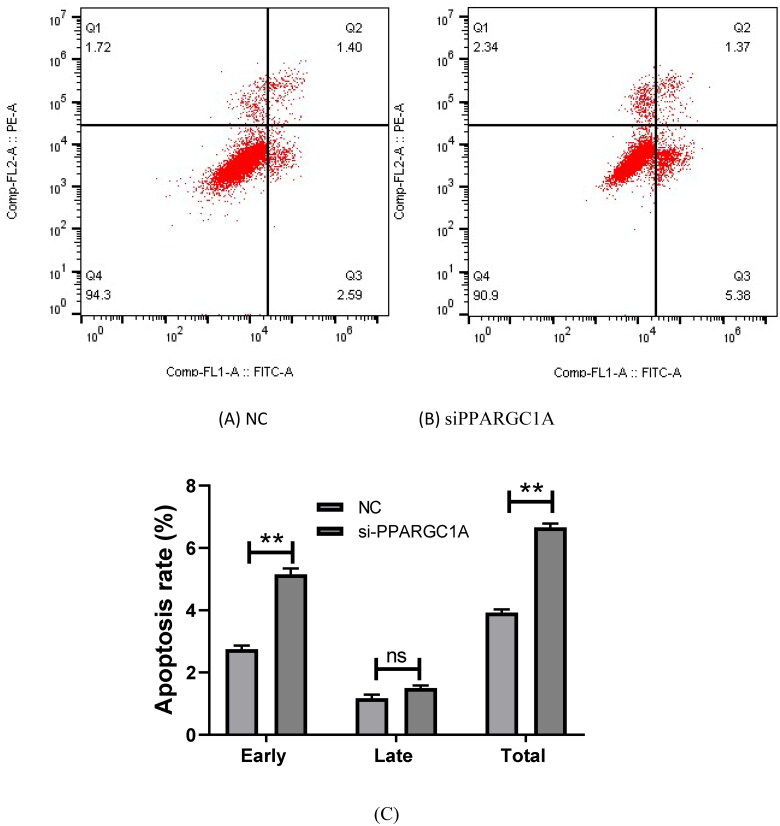
Effect of PPARGC1A gene knockdown on apoptosis in BuMECs. (A) (NC), and (B) (si-PPARGC1A) cell apoptosis distribution; (C) the rates of early, late, and total apoptosis

We examined apoptosis in BuMECs using flow cytometry. This analysis contributes to our understanding of the role of PPARGC1A in regulating apoptosis in mammary epithelial cells, shedding light on its potential involvement in cellular survival and programmed cell death pathways. We discovered that apoptosis in siPPARGC1A expression groups increased significantly in early and total stages relative to their respective negative control groups (*p* < 0.01) while having no significant effect in the late stage (Fig. 5).

### Impact of PPARGC1A gene on fatty acid secretion in BuMECs following RNAi

**Figure 6.**
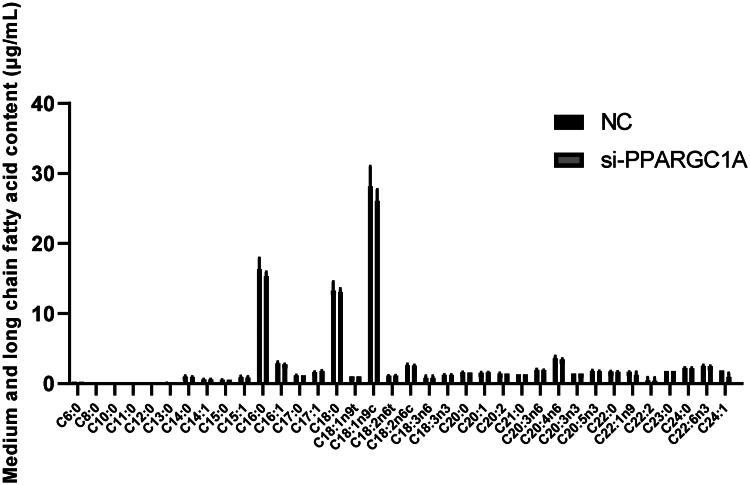
Effect of PPARGC1A gene on medium and long-chain fatty acids in BuMECs following RNAi Treatment.

To thoroughly investigate the function of the PPARGC1A gene in BuMECs, we studied its role in regulating 36 medium and long-chain fatty acids (Fig. 6). Our findings indicate that knocking down the PPARGC1A gene did not have significant effects.

### Impact of PPARGC1A gene knockdown on triglyceride secretion in BuMECs

**Figure 7.**
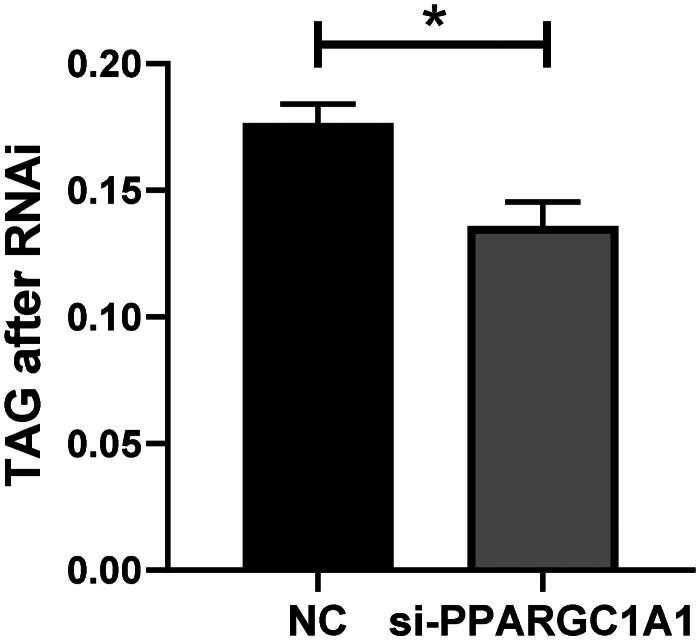
Impact of PPARGC1A gene knockdown on triglyceride secretion in BuMECs.

Following the knockdown of the PPARGC1A gene, triglyceride secretion levels in BuMECs were quantified and contrasted with control cells. The current study revealed a significant decrease in triglyceride secretion upon PPARGC1A RNAi expression in BuMECs (Fig. 7; *p* < 0.05). This finding clarifies the distinct role of the PPARGC1A gene in controlling triglyceride secretion in BuMECs, enhancing our comprehension of its involvement in lipid metabolism within mammary epithelial cells.

### Impact of PPARGC1A gene knockdown on β-casein concentration in BuMECs

**Figure 8.**
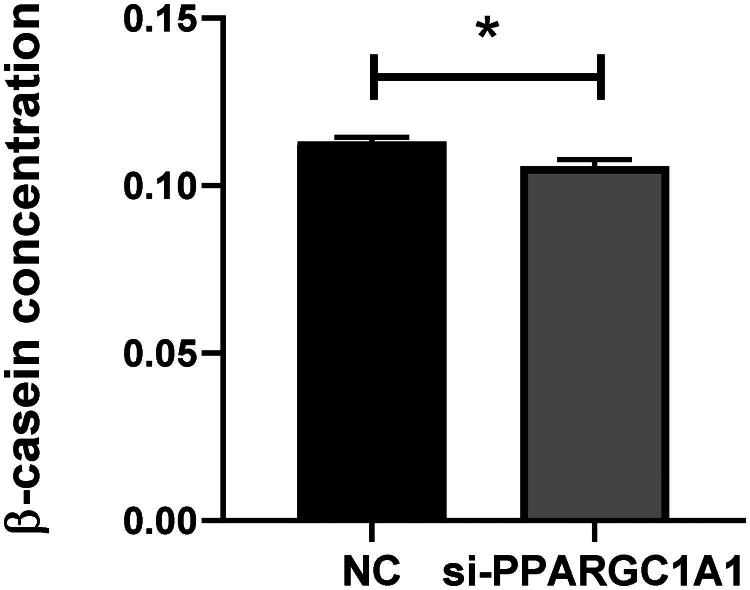
Impact of PPARGC1A gene knockdown on β-casein levels in BuMECs.

After the RNAi-mediated suppression of the PPARGC1A gene in BuMECs, β-casein levels were measured and contrasted with those in control cells. The objective of this study is to elucidate the effect of the PPARGC1A gene on β-casein production, a crucial component of milk protein synthesis. Our findings indicate a significant decrease in β-casein production in BuMECs following PPARGC1A gene knockdown (*p* < 0.05; Fig. 8). The use of the ELISA assay facilitated the evaluation of β-casein concentration changes consequent to PPARGC1A gene suppression, thereby shedding light on the gene’s regulatory role in β-casein production in BuMECs.

### PPARGC1A protein expression in BuMECs following RNAi Treatment

**Figure 9.**
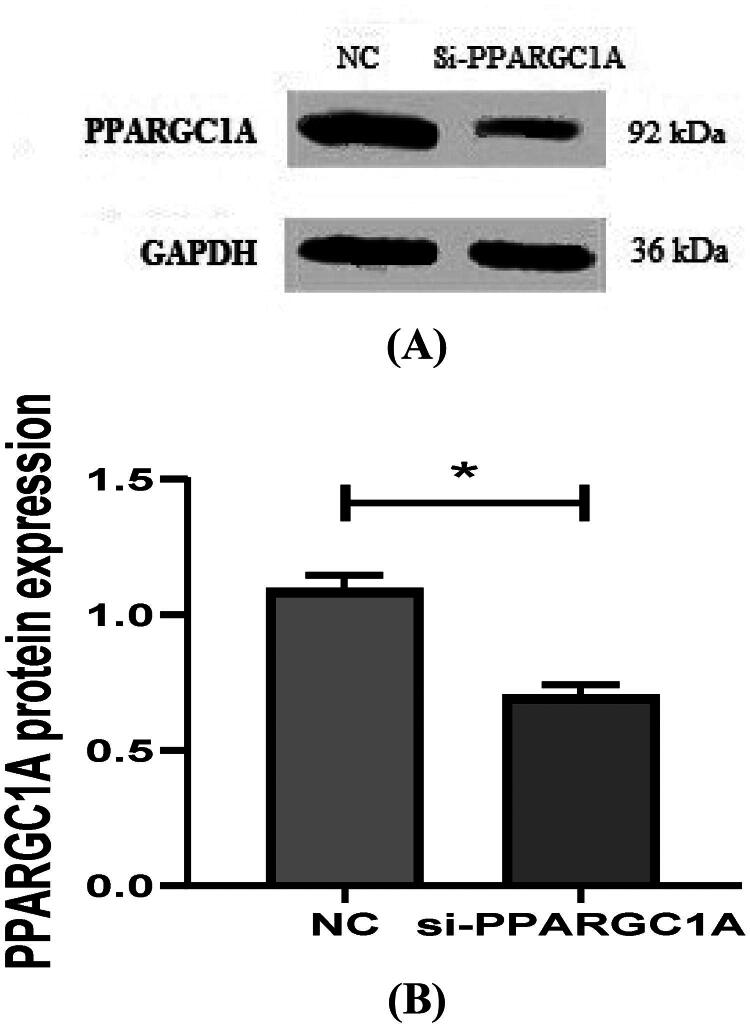
(A, And B) detection of protein expression of *PPARGC1A* gene in BuMECs after RNAi.

The protein expression of the *PPARGC1A* gene in BuMECs after RNAi was analyzed using Western Blotting, which helped us to find the lower levels of *PPARGC1A* protein in BuMECs following gene knockdown (*p* < 0.05; Fig. 9). By comparing the protein expression in RNAi-treated BuMECs to that in control cells, we could gain the impact of RNAi on the PPARGC1A protein levels, furthering our understanding of its function in mammary epithelial cells.

## Discussion

Understanding the genetic underpinnings that drive cell proliferation in mammary gland tissues is pivotal for improving lactation efficiency and milk yield.^17^ The PPARGC1A gene is known for its metabolic regulatory functions across various tissues.^18^ However, its involvement in the mammary glands, particularly buffalo, remains underexplored. To investigate the role of the PPARGC1A gene in regulating mammary epithelial cells in buffalo, we conducted a study on the effects of its knockdown on BuMECs. Gene knockdown is an experimental technique that reduces the expression of one or more genes and RNA interference (RNAi) occurs by the degradation of mRNA.^19^ Recently, mammary epithelial cells have proven to be an effective model for elucidating the physiological functions of the mammary gland, notably in studies of lipogenesis, cellular proliferation, casein production, and apoptosis synthesis.^17^ PPARGC1A plays a role in mitochondrial biogenesis and oxidative phosphorylation, both of which are crucial for supplying the energy necessary for cell cycle progression. This gene is particularly important in energy-demanding tissues like the heart, skeletal muscle, and mammary glands.^10^

In this study, we found that knocking down the PPARGC1A gene expression significantly impacted BuMEC growth, affecting the cell cycle, proliferation, and apoptosis. Notably, the cell population markedly increased at the G0/G1 stage and decreased during the S phase, with no significant change during the G2/M phase. During the G1 phase, where cell growth occurs, PPARGC1A is important for providing metabolic resources for cell growth. The knockdown of this gene may reduce cellular energy levels, potentially leading to a delayed G1/S transition as the cells may be unable to meet the metabolic demands for progressing to the next phase.^20^ In the S phase, where DNA synthesis occurs, PPARGC1A knockdown could affect the energy needed for DNA replication. Limited energy could slow S phase progression or cause DNA replication stress. The G2 phase involves preparation for mitosis, where complete DNA replication and repair are imperative. Reduced energy output due to PPARGC1A knockdown may hinder these processes. Mitosis requires considerable energy for cytoskeletal rearrangements and chromosome movements. PPARGC1A knockdown may thus compromise the cell’s energy state, potentially causing flawed mitotic events or activating cell cycle checkpoints and leading to arrest.^20^ Cell cycle arrest, prolonged due to energy insufficiency or unresolved DNA damage, might trigger apoptotic pathways. We observed that apoptosis significantly increased in both early and total stages without markedly affecting the late stage. The downregulation of PPARGC1A in mammary glands can profoundly influence apoptosis.^21^ This knockdown might disrupt the balance between cell proliferation and apoptosis, potentially leading to abnormal growth and function, which could impair lactation, reduce milk production, and compromise mammary gland health. Other studies have shown that PPARGC1A is involved in cell proliferation, differentiation, and apoptosis within the mammary gland; furthermore, it may inhibit these processes and regulate other biological functions such as cell migration, invasion, and neoplastic transformation.^22^ We conducted a cell viability assay to ascertain the role of PPARGC1A in cell proliferation. Our results indicated a significant decrease in both viability and cell number in BuMECs following PPARGC1A knockdown. As a pivotal regulator of metabolic pathways, diminished PPARGC1A expression can adversely affect glucose and fatty acid metabolism, integral to biosynthesis within the mammary gland. Cells with reduced PPARGC1A may be unable to fulfill the heightened energy requirements of milk production, leading to reduced cell viability. Additionally, PPARGC1A regulates genes involved in antioxidant defense; its downregulation may weaken a cell’s capacity to counteract oxidative stress, resulting in reactive oxygen species accumulation that can damage proteins, lipids, and DNA. Such oxidative damage impairs cell health and can prompt apoptosis.^23^ Rapid proliferation of mammary gland cells occurs during growth and lactation periods; lower PPARGC1A levels could disrupt cell cycle progression, curbing proliferation and impacting the maintenance of mammary gland tissue.^23^ Our findings implicate PPARGC1A knockdown in the decreased viability of mammary gland epithelial cells. Furthermore, our current study demonstrates that PPARGC1A gene knockdown notably impacts genes such as BAD, P53, and XDH, which are associated with cell cycle, apoptosis, and proliferation. BAD, as a pro-apoptotic member of the Bcl-2 family, may be indirectly upregulated due to the reduced energy environment stemming from PPARGC1A knockdown.^24^ The p53 tumor suppressor, responsive to metabolic changes caused by PPARGC1A knockdown, may result in G1 arrest or apoptosis.^25^ Our findings on the mRNA expression of related genes indicate a significant impact of p53 on BuMECs. XDH, involved in purine metabolism, and crucial for nucleotide synthesis and cell function,^26^ may also be influenced by decreased PPARGC1A activity, potentially hampering milk production by affecting cell proliferation and energy resources. Our findings revealed that the PPARGC1A gene regulates the growth of BuMECs, including proliferation, the cell cycle, and apoptosis.

In our Western Blot analyses, we observed that buffalo mammary gland cells exhibit a notable decrease in the expression of milk protein synthesis genes, such as caseins, upon PPARGC1A knockdown.^27^ This reduction may lead to lower milk protein content and could influence the quality and nutritional value of buffalo milk, which is vital for dairy production. Moreover, in the present study, we performed ELISA assays revealing significant negative effects of PPARGC1A on β-casein synthesis in BuMECs. Casein, comprising about 80% of milk protein, plays an essential role in defining milk quality.^28^ We uncovered STAT5 as having a significant influence on BuMECs. STAT5, a key player in prolactin signaling within mammary cells, facilitates lactogenesis.^29^ PPARGC1A may interact with prolactin-mediated pathways, with its knockdown potentially affecting STAT5 signaling, thereby leading to a reduction in milk protein gene expression and compromised development of mammary glands during lactation. Sharma et al.^30^ reported higher casein mRNA levels during early lactation, along with increased FABP3 expression, involved in milk fat synthesis, and sustained expression of regulatory genes STAT5 and SREBF1, highlighting their roles in lactogenesis. They established a positive link between PPARGC1A gene expression and casein concentration in milk, aligning with the established functions of PPARGC1A and providing new insights into the molecular regulation of milk protein synthesis. Our findings revealed that the PPARGC1A gene regulates protein synthesis in BuMECs.

Triglycerides (TGs), which make up about 95% of milk fat, are one of the key determinants of milk quality, influencing its taste and contributing fundamentally to its properties.^31^ In our previously conducted association studies,^7^ polymorphisms in the PPARGC1A gene were found to influence the fat percentage of buffalo milk, with the SNP g.286986 G > A being particularly associated with milk fat content. Additionally, our current research reveals a significant relationship between triglyceride secretion and PPARGC1A expression post-RNAi treatment in BuMECs. PPARGC1A has a pivotal role in lipid homeostasis regulation. Its downregulation in buffalo mammary glands significantly affects TG levels. Triglycerides are a primary source of energy and are instrumental in the synthesis of milk fat.^32^ The reduction in PPARGC1A expression suppresses genes involved in fatty acid synthesis and uptake, leading to decreased TG accumulation, with potential implications for milk composition and output in buffaloes.^14^ Bionaz and Loor underscore the importance of a gene network that regulates milk fat synthesis, highlighting the synergistic role of PPARGC1A with SREBP1 in dairy cows.^33^ Similarly, Zhou et al.^14^ observed that milk from high PPARGC1A expressers contained higher fatty acid concentrations, positing a correlation between PPARGC1A expression levels and milk fat content. The present study revealed that PPARGC1A knockdown significantly decreases triglyceride (TG) levels and affects the composition of certain fatty acids, suggesting that PPARGC1A plays a role in influencing the milk fat content of buffalo milk by modulating lipid synthesis in epithelial cells. Our research indicates that the downregulation of the PPARGC1A gene exerts a considerable impact on the expression of FABP3 and SREBF1, which are crucial in milk fat production. FABP3, or Fatty Acid Binding Protein 3, is essential for the intracellular transport of long-chain fatty acids and their derivatives. Given the involvement of PPARGC1A in energy metabolism, its suppression could potentially decrease FABP3 expression, leading to diminished uptake and use of fatty acids in mammary epithelial cells. SREBF1—or Sterol Regulatory Element Binding Transcription Factor 1—emerges as a vital transcription factor for activating genes that control lipid and triglyceride synthesis, influencing the expression of enzymes and proteins linked to milk fat synthesis like FABP3, thus impacting fatty acid synthesis and transport.^34^ It has been shown that silencing SREBP1 expression inhibits the expression of genes related to milk fat synthesis in BuMECs.^15^ Zhou et al.^35^ discovered that the overexpression of AGPAT6 in buffalo mammary epithelial cells significantly boosted the mRNA expression of the SREBF1 gene and considerably lowered that of XDH and PPARGC1A, but did not significantly affect the mRNA abundance of FABP3. In another study by Zhou et al.,^14^ the knockdown of the PPARG gene notably decreased the expression of FABP3 and PPARGC1A without impacting SREBF1 expression. They concluded that genes such as PPARG, INSIG1, PPARGC1A, INSIG2, and SREBF2 might interact in the expression of genes involved in buffalo milk fat synthesis.^14^ Bionaz and Loor^36^ underscored the synergistic relationship between PPARG, PPARGC1A, and INSIG1 in modulating the role and expression of SREBF1 in the context of milk fat synthesis in dairy cows. Our findings revealed that the PPARGC1A gene regulates lipogenesis in BuMECs.

This accumulation of insights affords opportunities for developing interventions aimed at adjusting cell growth and optimizing fat and protein content in milk. Such strategies can potentially augment the nutritional profile and health benefits of buffalo milk, contributing significantly to dairy production optimization and consumer health.

## Conclusion

Our findings position the PPARGC1A gene as a key regulatory element in the growth and development of mammary glands, highlighting its significance as a potential genetic marker for boosting milk production efficiency in water buffalo. Ongoing research into the intricate regulatory networks associated with PPARGC1A will prove advantageous in refining breeding practices and managing dairy production more effectively.

## Data Availability

The data used to support the findings of this study are included in the article.
